# Functional Foods for Cholesterol Management: A Review of the Mechanisms, Efficacy, and a Novel Cholesterol-Lowering Capacity Index

**DOI:** 10.3390/nu17162648

**Published:** 2025-08-15

**Authors:** Daniel A. Jacobo-Velázquez

**Affiliations:** Tecnologico de Monterrey, Escuela de Ingeniería y Ciencias, Campus Monterrey, Av. Eugenio Garza Sada 2501 Sur, Monterrey 64849, NL, Mexico; djacobov@tec.mx

**Keywords:** cholesterol-lowering foods, functional foods, nutraceuticals, LDL cholesterol, cardiovascular disease prevention, Cholesterol-Lowering Capacity Index (CLCI), personalized nutrition, food bioactives, food labeling, dietary interventions

## Abstract

Cardiovascular disease (CVD) remains the leading cause of death worldwide, with elevated low-density lipoprotein cholesterol (LDL-C) as a major risk factor. Beyond medications, dietary interventions and functional foods offer significant cholesterol-lowering potential. This article provides a comprehensive review of functional foods and nutraceutical ingredients that help to reduce cholesterol levels and introduces the novel Cholesterol-Lowering Capacity Index (CLCI), designed to quantify and communicate the efficacy of such foods. In doing so, it summarizes key functional components, including plant sterols/stanols, viscous fibers, soy protein, red yeast rice, berberine, polyphenols (e.g., bergamot extract, garlic), and others, highlighting their mechanisms of action and the typical LDL-C reductions observed in clinical studies. Strategies for the design of next-generation cholesterol-lowering foods are discussed, such as combining multiple bioactives for synergistic effects, personalized nutrition approaches, and novel food processing techniques to enhance bioavailability. Building on these strategies, the CLCI is then proposed as a practical scoring system, analogous to the glycemic index for blood sugar, that integrates the evidence-based potency of ingredients, effective dosing, and synergistic interactions into a single metric. A methodology for the calculation of the CLCI is presented, alongside potential applications in food labeling, clinical guidance, and dietary planning.

## 1. Introduction

Hypercholesterolemia (high blood cholesterol) is a well-established risk factor for atherosclerosis and cardiovascular disease (CVD). Epidemiological data have shown that lowering LDL cholesterol produces a proportional reduction in CVD risk [1,2]. Statin medications are effective in reducing LDL-C and cardiovascular events, but there is growing interest in non-pharmacological approaches such as diet modification and functional foods [3]. Clinical guidelines recommend lifestyle changes as a first-line strategy in managing elevated cholesterol, which can reduce LDL-C by roughly 10–20% in many individuals [2,4]. In this context, functional foods and nutraceuticals have emerged as promising adjuncts or alternatives to medication for cholesterol control [5,6].

Functional foods are broadly defined as foods that provide health benefits beyond basic nutrition. According to the European Commission’s consensus, a functional food is any food (not a pill) that has been formulated or enriched to confer a measurable health benefit when consumed as part of a normal diet [3]. Examples include foods fortified with bioactive ingredients or those naturally high in compounds that promote health. In the cholesterol-lowering arena, classic examples are margarine or dairy products enriched with plant sterols, which have a well-demonstrated LDL-C-lowering effect and have earned approved health claims in Europe and the U.S. [6]. The market for cholesterol-targeted functional foods and nutraceutical supplements has grown steadily over the past two decades, reflecting the public demand for “heart-healthy” foods and preventive nutrition strategies [5].

This article reviews the current knowledge of functional foods for cholesterol reduction and discusses innovative approaches to their design and utilization. It first examines the mechanisms by which various functional ingredients lower blood cholesterol, highlighting key examples and clinical evidence [7]. It then explores emerging strategies for the development of the next generation of cholesterol-lowering foods, including computational bioactive discovery, synergistic ingredient combinations, personalized nutrition, and novel food technologies that enhance efficacy and consumer acceptability [8]. Finally, it introduces the Cholesterol-Lowering Capacity Index (CLCI), a proposed unified metric to quantify the efficacy of functional foods and nutraceuticals in lowering cholesterol. By integrating scientific evidence into a practical score, the CLCI is intended to guide product development, support clinical dietary planning, and facilitate informed consumer choices. This framework aims to serve as a reference for food industry stakeholders and researchers working to combat hypercholesterolemia through evidence-based nutritional strategies.

## 2. Cholesterol and Cardiovascular Risk: Rationale for Functional Foods

Cholesterol plays vital physiological roles as a component of cell membranes and as a precursor for steroid hormones and bile acids. However, chronically elevated circulating cholesterol, particularly low-density lipoprotein cholesterol (LDL-C), greatly accelerates atherosclerotic plaque formation and increases cardiovascular risks. In addition to LDL cholesterol, cholesterol carried on triglyceride-rich remnant lipoproteins (such as very-low-density lipoproteins and intermediate-density lipoproteins) is also considered atherogenic and may contribute to plaque development. Large-scale trials have conclusively demonstrated that lowering LDL-C, whether through medications or dietary intervention, significantly reduces the incidence of cardiovascular events; for example, each 1 mmol/L reduction in LDL-C corresponds to an approximately 20% decrease in major vascular events [1,2].

Diet influences cholesterol levels by modulating the balance between input (dietary intake and hepatic synthesis) and output (excretion via bile and feces) [3]. Traditional cholesterol-lowering diets, characterized by low saturated fat and dietary cholesterol and high in dietary fiber, can improve lipid profiles, often achieving 10–20% reductions in LDL-C in individuals with elevated cholesterol levels [4,5]. Given that dietary changes are the first line of intervention in managing hypercholesterolemia, there is a strong rationale to further enhance this approach through the integration of functional foods.

Functional foods operate on the principle that specific nutrients or bioactive compounds can modulate distinct aspects of cholesterol metabolism. Certain ingredients inhibit cholesterol absorption in the intestines, others suppress endogenous synthesis in the liver, and some enhance LDL-C clearance or promote excretion. By incorporating effective doses of such components into everyday foods, the cholesterol-lowering potential of the diet can be amplified beyond that achieved through general healthy eating alone [7]. In this context, functional foods represent a bridge between conventional dietary strategies and pharmaceutical therapies, offering drug-like benefits within food matrices that can improve adherence and appeal to individuals seeking natural, non-pharmacological solutions for heart health. Unlike dietary supplements, which are typically consumed in capsule or powder form to complement the diet, functional foods deliver health benefits through the consumption of conventional food products. In contrast, medical foods are formulated for the dietary management of specific diseases and are intended to be used under medical supervision. This distinction clarifies the unique positioning of functional foods as accessible, preventive tools within everyday nutrition.

There is a growing body of evidence supporting the efficacy of functional foods and nutraceuticals in cholesterol management. For individuals with mild to moderate hypercholesterolemia, or those intolerant to statins, these foods offer safe and accessible LDL-lowering effects [3]. Regulatory bodies such as the U.S. Food and Drug Administration (FDA) and the European Food Safety Authority (EFSA) have approved health claims for components such as oat β-glucan and plant sterols based on clinical evidence of LDL-C reduction [6]. Notably, combining multiple cholesterol-lowering foods can produce additive or even synergistic effects, comparable to those of pharmaceutical interventions. In a landmark trial, Jenkins et al. [9] demonstrated that a dietary portfolio including plant sterols, viscous fibers, soy protein, and nuts reduced LDL-C by approximately 30%, an effect similar to that of a starting dose of statins. Real-world dietary patterns incorporating these foods have sustained LDL-C reductions of 10–20% over time [5], underscoring the utility of functional foods in comprehensive cardiovascular prevention strategies and reinforcing the importance of systematically designing and classifying such products.

## 3. Functional Foods for Cholesterol Reduction: Mechanisms and Examples

**Figure 1:**
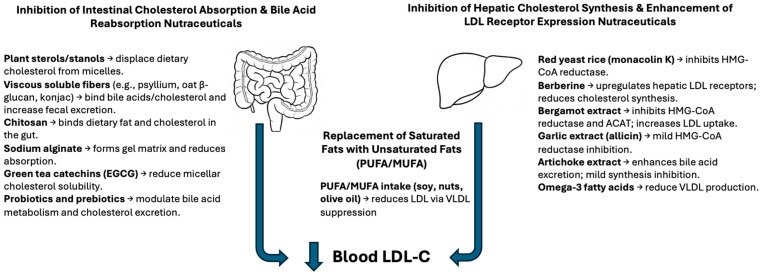
Mechanisms of action of cholesterol-lowering nutraceuticals. Schematic representation of the two main physiological targets for nutraceutical interventions: the intestine and the liver. Nutraceuticals such as plant sterols, soluble fibers, chitosan, alginates, and green tea catechins act in the intestine to reduce cholesterol absorption and increase fecal sterol excretion. In the liver, compounds like red yeast rice, berberine, garlic, bergamot extract, and unsaturated fatty acids reduce LDL-C by inhibiting cholesterol synthesis, enhancing LDL receptor activity, or modifying lipoprotein metabolism. These mechanisms converge to reduce circulating LDL cholesterol levels. Abbreviations: PUFA, polyunsaturated fatty acid; MUFA, monounsaturated fatty acid; EGCG, epigallocatechin gallate.

Cholesterol-lowering functional foods can be categorized by their primary mechanisms of action on cholesterol metabolism [10]. Figure 1 illustrates the major physiological targets that these foods act upon, and Table 1 summarizes key examples of ingredients, their sources, and their efficacy. This section discusses each category of action with examples and evidence.

### 3.1. Inhibition of Cholesterol Absorption

One of the most direct approaches is to prevent cholesterol from being absorbed in the gut. Plant sterols and stanols are quintessential agents in this category. These plant-derived compounds structurally resemble cholesterol and compete with dietary cholesterol for incorporation into mixed micelles in the intestine. By displacing cholesterol, sterols/stanols reduce the amount of cholesterol absorbed into the bloodstream. Regular consumption of ~2 g per day of plant sterols/stanols typically reduces LDL-C by about 7–12% [6,19]. Indeed, meta-analyses confirm an ~8–10% LDL reduction on average from such doses. Sterols are thus highly effective, and their cholesterol-lowering efficacy is relatively consistent across foods as long as a sufficient daily dose is provided (commonly achieved via fortified spreads, yogurts, milk, or capsules). While generally safe, very high intakes of sterols are not advised for individuals with sitosterolemia, a rare autosomal recessive disorder characterized by the impaired excretion of plant sterols and excessive absorption in the intestine. In affected individuals, dietary plant sterols can accumulate in tissues and contribute to premature atherosclerosis, making plant sterol intake potentially harmful. Some studies note that sterol supplementation can slightly lower beta-carotene levels (so maintaining a balanced diet is recommended) [6].

Another strategy to curb absorption is through viscous soluble fibers. Soluble fibers (like oat β-glucan, barley β-glucan, psyllium husk, pectin, and konjac glucomannan) form a gel in the intestinal lumen that binds cholesterol and bile acids, impeding their absorption and accelerating their excretion. This effectively reduces the circulating cholesterol pool. Clinical trials show that the daily intake of ~3 g of oat β-glucan can lower LDL-C by about 5–7%, and higher intakes of soluble fiber (e.g., 5–10 g/day, as in some psyllium regimens) can yield around 5–10% reductions in LDL-C [18]. For example, a meta-analysis of 28 trials found that 3 g/day of oat β-glucan reduced LDL by ~0.25 mmol/L (~6%) on average. Psyllium fiber (often taken as ~10 g/day from supplements or fiber-fortified foods) produces a similar LDL reduction of roughly 5–10% [17]. These fibers are most effective when consumed daily and with meals. Notably, the viscosity and molecular weight of the fiber influence its impact: more viscous fibers (like high-molecular-weight β-glucans) tend to have greater cholesterol-lowering effects than less viscous ones [18].

Several other compounds also reduce cholesterol absorption. Chitosan, a fiber-like polysaccharide from shellfish exoskeletons, can bind fat and cholesterol in the gut. Randomized trials of chitosan have shown modest LDL-C reductions (in the order of 5% or ~8–10 mg/dL) relative to placebos, indicating a small but significant effect (particularly in older adults) [11,12]. Algal fibers such as sodium alginate (from seaweed) have similar gel-forming and binding actions. In animal studies, soluble alginate markedly increased fecal sterol excretion, and, in human trials, alginate drinks prevented rises in serum cholesterol when subjects consumed a high-cholesterol diet [13,14]. Green tea catechins (especially in high doses or with specific structures like galloylated catechins) have been reported to mildly inhibit intestinal lipid emulsification and absorption. Meta-analyses of green tea consumption show a small LDL-C reduction (typically ~3–6%) in the short term, although their primary cardiovascular benefit may lie in antioxidant effects [16]. Overall, by targeting intestinal absorption, these functional ingredients lower the influx of cholesterol into the bloodstream.

### 3.2. Inhibition of Cholesterol Synthesis (and Enhanced LDL Clearance)

A second major mechanism is to inhibit the body’s own cholesterol production in the liver, which in turn can boost the removal of LDL from the blood. The most potent natural agent in this class is red yeast rice (RYR)—a fermented rice product that contains monacolin K, a compound that is chemically identical to the statin drug lovastatin. RYR has been used as a traditional remedy, and, at effective doses, it substantially lowers cholesterol. Clinical trials using RYR supplements providing about 5–10 mg/day of monacolin K have demonstrated LDL-C reductions in the range of 15–25% over ~2 months [26]. This magnitude approaches that of low-dose prescription statins, which is not surprising given the shared mechanism (HMG-CoA reductase inhibition). In fact, an international expert panel has recognized phytosterols and RYR as the nutraceuticals with the strongest lipid-lowering evidence [7]. RYR is often included in multi-ingredient “cholesterol support” formulations. However, because monacolin K is a statin analog, concerns exist about its safety and standardization; a 2018 EFSA opinion cautioned about potential side effects at high doses and variability in content between products [26]. Some countries regulate RYR supplements for these reasons. Nonetheless, at moderate doses, RYR appears safe for most and offers a powerful cholesterol-lowering option.

Another notable ingredient is berberine, a natural alkaloid from Berberis plant species. Berberine has a unique mechanism: it upregulates the expression of LDL receptors in the liver, thereby enhancing the clearance of LDL-C from the blood. It also modestly inhibits cholesterol synthesis and improves insulin sensitivity. A typical effective dosage is around 1 g per day (often split into two doses). Trials of berberine have consistently found significant LDL-C reductions. For example, one study reported a ~24% reduction in LDL after 2 months of 500 mg twice daily [27]. Meta-analyses indicate that berberine can lower LDL-C by approximately 10–15% (with larger drops in some cases) [7,28]. In patients with metabolic syndrome or diabetes, berberine also improves triglycerides and blood glucose, making it a valuable multifunctional nutraceutical. Importantly, berberine is well-tolerated; its side effects are minor (mainly gastrointestinal at higher doses) [29]. This favorable safety profile and oral efficacy have spurred interest in incorporating berberine into functional foods (e.g., in bars or drinks) for cholesterol management.

Various polyphenol-rich plant extracts also act to reduce hepatic cholesterol synthesis or otherwise improve lipid metabolism. A standout example is bergamot (*Citrus bergamia*) extract. Bergamot orange from Calabria is rich in unique flavonoids (such as brutieridin and melitidine) that have statin-like effects in the liver. Studies indicate that bergamot polyphenols can inhibit HMG-CoA reductase (the cholesterol-producing enzyme targeted by statins) and also ACAT (involved in cholesterol esterification), while additionally upregulating LDL receptors [7]. Bergamot’s multifaceted action (reducing cholesterol production, enhancing LDL uptake, and possibly reducing absorption) translates to significant lipid improvements. A recent systematic review of 14 randomized controlled trials found that bergamot extract (at doses of 500–1500 mg polyphenols per day) produced statistically significant reductions in total cholesterol, LDL-C, and triglycerides, along with increases in HDL-C [30]. The extent of LDL-C reduction varied among studies, ranging from ~8% in some trials to over 30% in others, with an average drop of around 15% at typical doses. This positions bergamot as one of the most effective botanical nutraceuticals for cholesterol. Moreover, short-term trials report a good safety profile with negligible side effects. Given its efficacy, bergamot polyphenols are being incorporated into experimental functional products (for instance, yogurt drinks or teas infused with bergamot extract) to capitalize on their cholesterol-lowering potential.

Other herbal extracts with mild cholesterol synthesis-inhibitory or LDL-lowering effects include garlic and artichoke leaf. Garlic (*Allium sativum*) contains organosulfur compounds (e.g., allicin), which are thought to interfere with cholesterol synthesis in the liver. Multiple studies and meta-analyses of garlic supplementation show modest cholesterol reductions—typically around 5–10% in LDL-C (roughly 8–9 mg/dL) over 1–3 months. A meta-analysis by Ried et al. [31] found significant but modest lowering of total and LDL cholesterol with garlic powder supplements, confirming its mild efficacy. Artichoke leaf extract (*Cynara scolymus*) has also demonstrated small but significant cholesterol-lowering effects. Artichoke is believed to enhance bile acid excretion and inhibit cholesterol synthesis via compounds like cynarin. Meta-analyses report that artichoke extract can reduce LDL-C by 10–15 mg/dL, translating to roughly a 5–8% decrease in LDL [32]. These effects, while not large, are statistically significant and contribute to overall lipid improvement. Both garlic and artichoke are thus considered useful adjuncts to the diet, albeit not potent enough to be sole therapies for high cholesterol. They exemplify the broader category of botanical nutraceuticals that offer mild LDL reductions alongside other health benefits (garlic also slightly lowers blood pressure; artichoke has antioxidant effects).

### 3.3. Replacement of Harmful Nutrients and Dietary Patterns

Functional foods can improve cholesterol levels not only by adding beneficial ingredients but also by replacing less healthy components of the diet. A prime example is modifying the dietary fat profile. Diets high in saturated fatty acids (SFAs) raise LDL-C, whereas replacing saturated fats with unsaturated fats (monounsaturated or polyunsaturated) leads to LDL-C reductions [22]. This is the rationale behind cholesterol-lowering margarines, spreads, or dairy alternatives rich in unsaturated fats. In a classic study, a diet high in monounsaturated fat (MUFAs, e.g., olive oil, nuts, avocado) lowered LDL-C by about 14% compared to a diet high in saturated fat, performing as well as the standard low-fat diet recommended for cholesterol. Specifically, Kris-Etherton et al. [33] showed that switching from a typical Western diet to a high-MUFA diet reduced LDL by ~14% and total cholesterol by ~10%. Mechanistically, diets richer in unsaturated fats may reduce hepatic VLDL production (since unsaturated fats are preferentially oxidized for energy, rather than being used for cholesterol synthesis) [23].

Many functional foods leverage this principle: for instance, products formulated with soy or other plant proteins instead of animal protein can lower the intake of saturated fat and dietary cholesterol. Soy protein itself exerts a small, intrinsic cholesterol-lowering effect (possibly via bioactive peptides and isoflavones affecting hepatic cholesterol metabolism), in addition to displacing animal fat. As noted by Jenkins et al. [34], about 25 g of soy protein per day can reduce LDL by roughly 7–10% when it replaces animal protein. This has led to the FDA recognition of soy protein’s heart health benefit (although the effect size is modest) [24]. In practice, introducing high-MUFA foods (e.g., nuts, olive oil-based spreads) or soy-based foods in place of butter, red meat, or dairy fat can meaningfully improve one’s lipid profile.

Omega-3 fatty acids (from fish oil or algae oil, e.g., EPA and DHA) are another dietary component often added to foods (like omega-3-enriched eggs, breads, or milk) for heart health. Omega-3s are better known for lowering triglycerides, but they can also have a slight LDL-lowering or neutral effect in many people. High doses of omega-3 (2–4 g/day as in prescription fish oils) tend to reduce triglycerides substantially and may raise LDL slightly in some cases; however, in hypertriglyceridemic individuals, they often lower the atherogenic small dense LDL. A recent meta-analysis of ~90 trials found that omega-3 supplementation produced a dose-dependent decrease in triglycerides and a modest reduction in non-HDL cholesterol [21]. The net impact of omega-3-fortified foods on LDL is usually minimal, but they contribute to cardiovascular risk reduction through triglyceride-lowering and anti-inflammatory effects. Thus, while omega-3-enriched functional foods might not score highly for LDL reduction per se, they are often included in heart-healthy diets for their indirect benefits.

Importantly, these replacements tie into overall dietary patterns. A functional food will have the greatest impact when used as part of a holistic dietary pattern improvement. For example, the Mediterranean diet (rich in olive oil, nuts, fish, and vegetables) inherently includes many of the elements discussed, namely unsaturated fats, fibers, and phytochemicals, which collectively yield favorable lipid profiles [5]. The portfolio diet is another clear demonstration: it combines multiple cholesterol-lowering foods (sterols, fibers, soy, almonds) in one diet. Each component provides a modest benefit, but, together, they achieved an LDL reduction of ~30% in controlled studies [35]. Even outside research settings, adopting a diet that incorporates several functional foods can lead to significant cholesterol improvements (commonly 10–20% LDL reductions in practice). This synergistic effect has inspired food product developers to create single items that blend multiple bioactive ingredients. For instance, one might formulate a “heart-healthy” smoothie or bar that contains plant sterols, a viscous fiber, and plant protein, enabling consumers to conveniently obtain a cocktail of cholesterol-lowering components in one serving. Overall, replacing harmful nutrients (like saturated fat) with beneficial ones, and encouraging diverse diets, is a cornerstone of functional food strategies for cholesterol management.

### 3.4. Additional Functional Ingredients with Cholesterol Benefits

Beyond the major players above, there are other foods and bioactives that confer cholesterol benefits that are worth noting.

**Nuts and Seeds:** Almonds, walnuts, flaxseeds, and similar nuts are nutrient-dense foods high in unsaturated fatty acids, fiber, and phytochemicals. Regular consumption of ~30–50 g of nuts per day is associated with modest LDL-C reductions (often in the order of 5–7%) [5,33]. Nuts also consistently show cardiovascular outcome benefits in studies, likely due to a combination of lipid-lowering, anti-inflammatory, and antioxidant effects. They are essentially “natural” functional foods. Many cholesterol-lowering diets include a daily handful of nuts, and products like almond-enriched cereals or flaxseed-fortified breads leverage these benefits.

**Polyphenols and Antioxidants:** Foods rich in polyphenols (green tea, cocoa, berries, red wine, etc.) may not dramatically lower LDL-C levels, but they improve other aspects of cholesterol metabolism and vascular health. For example, polyphenols can reduce the oxidative modification of LDL particles, making LDL less atherogenic, and they can improve endothelial function. Berries in particular have been studied: adding strawberries or blueberries to a cholesterol-lowering diet further protected LDL from oxidation and improved HDL function without significantly changing the LDL concentration. These antioxidant effects contribute to risk reduction, even if LDL-C per se is unchanged. Innovative functional foods might include antioxidant-rich ingredients (like berry extracts, grape polyphenols, or cocoa flavanols) alongside primary cholesterol-lowering actives to provide comprehensive cardioprotective effects [9]. For instance, a yogurt or smoothie might combine plant sterols for LDL lowering and berries for antioxidant support.

**Probiotics and Prebiotics:** The gut microbiome can influence cholesterol metabolism (certain gut bacteria help to deconjugate bile acids or produce metabolites, affecting lipid levels). Some probiotic strains—particularly *Lactobacillus* and *Bifidobacterium* species—have been reported to yield small LDL-C reductions in clinical trials [36]. They may work by binding cholesterol in the gut or by producing acids that inhibit hepatic cholesterol synthesis. Likewise, prebiotic fibers (like inulin, fructooligosaccharides) can foster a microbiome that favors cholesterol excretion. While the cholesterol effects of probiotics/prebiotics are generally modest (a few percentage points of LDL reduction at best) and variable, their inclusion in functional foods (e.g., fermented dairy with added probiotic cultures) is a growing area of interest. Research is ongoing to identify more effective strains and synbiotic combinations [36] that could enhance cholesterol lowering.

**Combination Products:** As alluded to earlier, combining ingredients can amplify results. Some dietary supplements already market combinations: for example, Armolipid Plus (a supplement) contains red yeast rice, policosanol, berberine, folic acid, and astaxanthin. In clinical use, such combinations have been shown to lower LDL by 20% or more, comparable to a low-dose statin [7]. This synergy arises because each component tackles cholesterol via a different mechanism (one blocks absorption, another inhibits synthesis, etc.), yielding an additive impact. A functional food equivalent might be a beverage or bar that similarly unites multiple bioactives. Preliminary studies on multi-ingredient functional foods are promising. For instance, a pilot trial of a yogurt fortified with plant sterols, viscous fiber, and antioxidants found greater LDL reductions than with any single component alone. These examples underscore that synergistic formulation is a key concept: the whole can be greater than the sum of its parts in cholesterol-lowering functional foods.

## 4. Innovative Approaches in Functional Food Design for Cholesterol Reduction

The field of functional foods is rapidly evolving, moving beyond simply “add a proven ingredient to a food” toward more advanced design and integration strategies. Several innovative approaches are shaping the next generation of cholesterol-lowering foods.

### 4.1. Evidence-Based Formulation and Computational Bioactive Discovery

Developers are increasingly using computational tools and bioinformatics to guide the discovery and combination of cholesterol-lowering food components. This approach treats functional food development akin to drug discovery. Researchers first identify molecular targets involved in cholesterol metabolism (such as intestinal transporters or hepatic enzymes like HMG-CoA reductase, NPC1L1, PCSK9, ACAT, etc.) and then screen libraries of natural compounds in silico to find those that interact with these targets. For example, Azevedo et al. [8] virtually “docked” numerous phytochemicals into key proteins of cholesterol metabolism and identified novel inhibitors and activators from foods. Such computational modeling can predict that certain plant molecules (perhaps a flavonoid or terpenoid) will bind to a cholesterol-regulating enzyme. These predictions are then tested in the lab (in vitro) and eventually in animal or human studies. This strategy has led to promising discoveries—for instance, specific phenolics that block the intestinal cholesterol transporter NPC1L1, or natural terpenes that upregulate LDL receptors in the liver. By elucidating structure–activity relationships, computational research also explains why some compounds are more potent, e.g., why phytosterols with certain side-chain lengths block absorption better, or how slight chemical modifications to a citrus flavonoid might enhance its HMG-CoA reductase inhibition. Armed with these insights, companies can rationally design food products by selecting or enriching the most effective bioactive compounds. We can envision, for example, formulating a “cholesterol-blocking beverage” by first using computer simulations to identify an optimal combination of natural inhibitors of cholesterol absorption and synthesis, rather than relying on trial-and-error mixing. This evidence-based formulation accelerates innovation and may uncover new food-based solutions that approach the efficacy of drugs. While still an emerging area, early successes suggest that integrating nutritional science with computational chemistry provides a powerful toolkit for precision functional food design [8].

### 4.2. Personalized Nutrition and Tailored Functional Foods

Another important trend is personalization. Individuals respond differently to dietary interventions due to genetic differences (nutrigenetics), gut microbiome variability, and other factors. A one-size-fits-all functional food may underperform in certain people but work very well in others. Thus, a forward-thinking approach is to tailor functional food recommendations, or even the formulations themselves, to specific subgroups of people for the maximum benefit. For example, about 20% of people are “hyper-absorbers” of cholesterol (their bodies absorb an unusually high fraction of dietary cholesterol). Such individuals may benefit enormously from functional foods that target absorption (like sterol-fortified foods or fibers), since blocking absorption addresses their particular issue. On the other hand, some people are “hyper-synthesizers”, who overproduce cholesterol in the liver; they might respond better to ingredients that inhibit synthesis (like red yeast rice or bergamot extract). Researchers are identifying genetic markers that predict responses—for instance, polymorphisms in the APOE gene or the intestinal transporter gene NPC1L1 can influence how well someone’s LDL drops with plant sterols or low-fat diets [37]. In the near future, a consumer might take a simple genetic test and find out that they are, for example, an APOE4 carrier who would particularly benefit from increasing viscous fiber and avoiding high saturated fat for cholesterol control. Similarly, the gut microbiome plays a role: certain gut bacteria produce metabolites (like secondary bile acids) that affect cholesterol levels. If a person’s microbiome composition is known, one could tailor prebiotic or probiotic functional foods to them—for example, giving a specific probiotic strain to enhance bile acid deconjugation in someone whose microbiome lacks this. While personalized functional foods are still in early development, this approach represents a shift from mass market products to targeted nutrition solutions. Even simply segmenting advice, such as recommending a sterol-fortified yogurt to one patient but a soy protein bar to another based on their metabolic profiles, could increase the overall effectiveness of diet therapy for hypercholesterolemia. Personalization maximizes the LDL reduction each individual can achieve and could improve user engagement, as people receive interventions suited to their unique biology.

### 4.3. Novel Food Vehicles and Delivery Technologies

How a bioactive ingredient is delivered in a food matrix can greatly influence its efficacy. New food processing and formulation technologies are enabling the incorporation of cholesterol-lowering compounds into a wider range of products, in forms that optimize their bioavailability and consumer appeal. A key development is microencapsulation, i.e., packaging microscopic droplets or particles of a bioactive in a protective coating (e.g., a polysaccharide or protein matrix). Microencapsulation allows sensitive or hydrophobic ingredients to be added to foods without compromising stability or taste. For instance, originally, it was challenging to add plant sterols to low-fat foods because sterols are fat-soluble and would crystallize or taste chalky. Now, microencapsulated sterols can be dispersed in beverages like orange juice or skim milk seamlessly [38]. Similarly, fish oil (omega-3) can be encapsulated to prevent oxidation and a fishy taste, enabling its addition to breads or snacks. Encapsulation can also mask strong flavors (as with garlic extract) and protect compounds from heat or light degradation during cooking and storage. Another innovation is the use of nanotechnology, creating nanoemulsions or nanoparticles that improve the solubility and absorption of compounds like curcumin or certain polyphenols. These ultra-small carriers can enhance the intestinal uptake of bioactives, potentially boosting their effectiveness at lower doses [39].

A futuristic approach in this realm is 3D food printing and structural design. Emerging 3D food printers can layer ingredients in precise configurations, which might control the release profile of nutrients in the digestive tract. One could design, for example, a high-fiber, multi-layered snack bar where a layer containing plant sterols is programmed to release in the small intestine (for absorption blocking) and another layer with a probiotic or enzyme releases in the colon. This spatial and temporal targeting within the GI tract could optimize where each component exerts its effect. Although in its infancy, 3D printing technology suggests a future of custom-structured functional foods tailored for maximal efficacy and even personalized to an individual’s needs [40].

We also see innovation in fermentation and biofortification techniques. Fermentation can be used to naturally enrich foods with cholesterol-lowering compounds: for instance, certain probiotic fermentations of dairy or soy can produce peptides or short-chain fatty acids during the process that have cholesterol-lowering actions (beyond the probiotics themselves) [41]. Traditional fermented foods (like kefir, kimchi, miso) are being studied for such effects. Meanwhile, agricultural biofortification is creating raw materials that are inherently rich in functional compounds, e.g., breeding oats to have higher β-glucan content or engineering plants to produce more stanols or omega-3s. These upstream innovations provide ingredients that naturally carry cholesterol-lowering power, simplifying the formulation of functional end-products.

### 4.4. Combining Efficacy with Sustainability and Acceptability

As new cholesterol-lowering foods are developed, it is increasingly important to address sustainability and consumer acceptance. Many nutraceutical ingredients are sourced from plants, algae, or other natural resources, so sustainable sourcing and production are key considerations. An innovative angle is utilizing food industry byproducts or underused resources as sources of functional compounds. For example, citrus peels (a byproduct of juice production) are rich in pectin (a soluble fiber) and flavonoids; extracting these not only provides a cost-effective functional ingredient but also reduces waste [42]. Similarly, algae and seaweed are fast-growing and can be harvested sustainably for compounds like omega-3 fatty acids, sterols, and viscous fibers (algal β-glucan) [43]. These can serve as eco-friendly alternatives to traditional sources (fish oil, plant sterols from soy, etc.). Emphasizing sustainability may also increase consumer acceptance, as modern consumers often prefer products that are natural, ethical, and environmentally conscious [44].

From a consumer standpoint, no matter how efficacious a functional food is, it must be palatable and convenient to have a real impact. Therefore, product designers focus on sensory optimization, ensuring that added ingredients do not adversely affect the taste, aroma, or texture. For instance, adding a high dose of psyllium fiber to a beverage can render it thick or gritty, so technological adjustments (like using a smaller particle size or a clear soluble fiber) are needed to maintain a pleasant mouthfeel. There is also behavioral design, which involves packaging and marketing strategies to encourage regular consumption (since consistency is key for cholesterol management). One concept is “stealth health”—integrating cholesterol-lowering ingredients into staple foods that people already eat daily, so that they obtain the benefits without extra effort. Japan’s FOSHU (Foods for Specified Health Use) program has many examples—ordinary foods like tea, soup, or rice fortified with functional ingredients (e.g., tea with added sterols, noodles with added fiber) that fit seamlessly into traditional diets [5]. In Western markets, similarly, orange juice fortified with plant sterols and breads baked with oat fiber are available. By exploiting common foods, the reach of functional ingredients can be vastly increased without requiring consumers to change their meal patterns drastically.

Finally, an often overlooked aspect of innovation is regulatory and scientific validation. To market a functional food with a health claim (such as “lowers cholesterol”), companies must generate rigorous evidence and navigate approval processes. Innovative design thus includes planning for clinical trials to prove efficacy in humans and monitoring long-term safety. For example, while plant sterol margarines and oat cereals have long-standing approved health claims, a novel combination bar (e.g., with bergamot extract and fiber) would need robust clinical data for regulatory approval and consumer trust. Encouragingly, regulatory bodies are increasingly recognizing the role of functional foods in health; recent guidelines in Europe and the US acknowledge that certain nutraceuticals can be part of dyslipidemia management [3]. Nonetheless, developers must also address any safety questions, e.g., high-dose nutraceuticals could have off-target effects. There have been isolated concerns, such as extremely elevated plant sterol levels being linked to CVD in rare cases, or liver enzyme elevations in a few patients on certain high-potency supplements [45]. Ongoing research and surveillance are needed to ensure that these products remain safe when used long-term by the general public. In summary, responsible innovation means not only maximizing LDL reduction but doing so in a way that is sustainable, palatable, evidence-based, and safe for consumers over the long term.

After reviewing the current landscape of functional ingredients and innovative design strategies, the next challenge lies in quantifying and comparing the cholesterol-lowering efficacy of these diverse foods and supplements. The following section introduces a proposed standardized index to address this need.

## 5. Proposed Index for Cholesterol-Lowering Capacity of Foods and Nutraceuticals

Despite the myriad functional foods and ingredients available for lowering cholesterol, there is currently no unified way to compare their potencies or to communicate their overall impacts to consumers and health professionals. To address this, herein, the Cholesterol-Lowering Capacity Index (CLCI) is proposed—a standardized score that quantifies how effective a given food or nutraceutical product is in reducing LDL cholesterol. This concept is inspired by analogous indices in nutrition, such as the glycemic index (which ranks carbohydrate foods by blood sugar response) and the ORAC score for antioxidant capacity. The CLCI would integrate multiple factors: the presence and levels of proven cholesterol-lowering compounds, the evidence-based efficacy of these compounds (from clinical trials or meta-analyses), their bioavailability in the given food matrix, and any synergistic interactions when multiple ingredients are combined. The result would be a single scale (for example, 0 to 100, or a categorical rating like “low/moderate/high capacity”) that can be assigned to a food or supplement. In principle, a higher CLCI score would indicate a greater expected LDL-C reduction when the item is consumed regularly as directed.

The CLCI is envisioned as a practical tool with several applications. For industry, it could be used in product development and labeling; companies could formulate products to achieve a target CLCI score and communicate this “score” on packaging for marketing and educational purposes. For clinicians and dietitians, the index would help in recommending dietary strategies (e.g., guiding patients to choose higher-CLCI foods or to combine items for a daily target score). For researchers, it offers a way to quantitatively compare interventions: for instance, a clinical trial of a new functional food could report how its CLCI score correlates with LDL changes, facilitating comparisons across studies. Overall, by translating complex nutritional data into an accessible metric, the CLCI aims to bridge scientific evidence and practical implementation in cholesterol management.

### 5.1. Key Nutraceutical Components and Scoring Basis

The foundation of the CLCI is the range of key nutraceutical components that have been shown to lower cholesterol. Drawing from the review above, the index would focus on ingredients with robust clinical evidence of LDL-C reduction. Each such component would be assigned a base score contribution corresponding to its typical cholesterol-lowering potency (as per an effective dose). Table 1 (presented earlier) lists many of these compounds and their efficacy. In constructing the index, we incorporate the following.

**Plant Sterols/Stanols**: Average LDL reduction ~10% for 2 g/day. These would merit one of the higher base scores (since few food ingredients match this efficacy). For example, if we normalize scores such that a 10% LDL reduction = 10 points, sterols might contribute ~10–12 points per standard serving, which provides ~2–3 g sterols [6,19].

**Viscous Soluble Fiber**: LDL reduction ~5–7% for ~3 g β-glucan or 10 g psyllium. Fibers might thus contribute approximately five points per effective dose in a product. If a product has half the effective dose, it receives ~2–3 points [17,18].

**Red Yeast Rice (Monacolin K)**: LDL reduction ~15–25% at typical supplement doses. This high potency would give it a base score within the 15–20 point range for a full dose (although the regulatory status as a “food” ingredient may vary) [7,26].

**Berberine**: LDL reduction ~10–15% at ~1 g/day. Base score ~10 points for a full 1 g serving (smaller amounts scale down accordingly) [27,28].

**Soy Protein/Isoflavones**: LDL reduction ~3–7% for ~25 g soy protein. Base score on the lower side (e.g., ~3–5 points) for a serving with high soy protein [24,34].

**Bergamot Extract**: LDL reduction ~8–15% at common doses. Base score may be ~10 points for a standard dose of bergamot polyphenols [30].

**Garlic Extract**: LDL reduction ~5% (up to 10%). Base score ~3–5 points for a full daily dose (e.g., ~0.5–1 g garlic extract) [31].

**Artichoke Extract**: LDL reduction ~5%. Base score similarly low, ~3–5 points for a typical dose [32].

**Omega-3 (high dose)**: Primarily for triglycerides; the LDL effect is neutral or slight. Likely not scored unless the product is specifically targeting TG [21].

**Others (nuts, green tea, etc.)**: Modest effects; if a food inherently contains them, they might add a small effect (e.g., a few points for a serving of almonds or high-EGCG tea, reflecting a minor LDL benefit) [5,9].

The idea is that each product can be analyzed for its content of these proven agents, and points are allocated proportionally. An effective dose (based on clinical trials) of a given component might be set as contributing a fixed number of points (anchored to its % LDL reduction). If a product has more or less than the effective dose, the score for the given component is scaled accordingly. For instance, if 2 g of plant sterols = 10 points, then 1 g sterol in a serving would contribute ~5 points. If 10 g of fiber = 5 points, then 5 g fiber = 2.5 points, and so on.

Importantly, these base scores must be evidence-based, derived from published studies and meta-analyses (ensuring that the index is grounded in science). Periodic updates would be needed as new research might show different efficacies, or as new nutraceuticals emerge.

### 5.2. Accounting for Bioavailability and Food Matrix

A critical aspect of the CLCI is adjusting scores according to how bioavailable and effective a compound is in the form in which it is consumed. Not all milligrams are equal; the cholesterol-lowering impact of a nutrient depends on how much of it is absorbed or active at the target site, which in turn can depend on the food matrix and processing. For example, plant sterols have high efficacy when eaten with some fat (to stimulate micelle formation); a sterol-fortified food that is fat-free might deliver less benefit than the same dose in a spread. Similarly, the release profile of a fiber (e.g., whole oat groats vs. isolated β-glucan in a drink) could affect the viscosity and bile binding in the gut. The CLCI scoring would therefore include bioavailability coefficients or modifiers. Practically, this means that, if a food form is known to diminish an ingredient’s effect, the score is discounted. For instance, if research [38] shows that phytosterol esters in a yogurt reduce LDL slightly less efficiently than in a spread, the index for a yogurt with 2 g sterols might be a point or two lower than 2 g in a spread. On the other hand, technologies like microencapsulation that improve stability or absorption might allow a full score [39]. Each category of ingredient could have guidelines, e.g., sterols in a high-fat matrix—100% bioavailability factor; sterols in low-fat matrix—80% factor, etc., based on absorption studies. These adjustments would ensure that the index reflects real-world efficacy rather than just nominal content.

Another example is as follows: monacolin K (from red yeast rice) might be fully active in a supplement pill, but, if added to a heat-processed food, some potency might be lost. The index would account for this by reducing its score contribution unless stabilization methods are used. Release and timing are also considered: a fiber that is highly fermentable in the gut might not bind as much bile, and different fibers could have different effectiveness scores, even at equal grams [18]. While these nuances add complexity, incorporating bioavailability factors makes the CLCI more accurate. It effectively rewards formulations that deliver ingredients in an optimal way and penalizes those that, by virtue of their formats or additives, have a reduced impact.

### 5.3. Incorporating Synergy (Combination Effects)

One of the most novel and valuable aspects of a composite index, and a major rationale for the CLCI, is capturing synergistic effects when multiple cholesterol-lowering components are present together. Often, using a combination of nutraceuticals yields a greater cholesterol reduction than would be expected from any single component alone. This is because different compounds act on different pathways (absorption, synthesis, etc.), complementing each other. For example, a sterol and a fiber together can have an additive LDL-lowering impact, and studies like those of Jenkins et al. [35] or Lin et al. [20] (in hamsters) have shown that the combined effects of certain ingredients can exceed the sum of each alone. Such synergy may also help to overcome endogenous counter-regulatory mechanisms that maintain cholesterol homeostasis, as demonstrated in pharmacological approaches where targeting multiple pathways, such as combining statins with PCSK9 inhibitors, achieves superior lipid-lowering outcomes. The CLCI should reflect this by giving a modest “bonus” when a product contains multiple complementary agents.

Herein, introducing a synergy factor in the index is proposed. Operationally, if a product contains at least two (or more) different classes of active ingredients, a small additional score is added to account for their synergy. For instance, suppose that a cereal has plant sterols (absorption inhibitor) and psyllium (bile sequestrant). Each has its base points, but, together, they might achieve a slightly greater LDL reduction than their sum. Thus, for example, a 10–20% bonus might be added to the combined score. The exact value could be calibrated from studies of combinations (some evidence suggests that a combination can yield a ~5 percentage point greater LDL reduction than expected). For simplification, the CLCI might add a flat +2 points for each distinct mechanism category beyond the first, or a multiplier if certain combinations are present. For example, if a product has components from three categories (e.g., sterols, fiber, and polyphenols), it could receive a larger synergy boost than one with just two components.

Synergy scoring would be conservative to avoid over-claiming—it might be capped at a certain number of extra points. The presence of synergy means that the index encourages portfolio-style products and communicates their value. It reinforces the concept that a diverse formulation is beneficial. From the consumer perspective, a high CLCI score would often imply that the product has multiple active ingredients working together, rather than just one in isolation.

### 5.4. Methodology for Calculating the CLCI

Bringing the elements together, the CLCI for a given food/supplement would be calculated through a step-by-step algorithm.

**Ingredient Identification:** List all cholesterol-lowering active components in one serving (or daily recommended serving) of the product. For each, note the amount present.

**Base Score Assignment:** For each component, assign a base score corresponding to its expected LDL reduction at that amount. This uses a reference database, e.g., X mg of ingredient Y = Z% LDL reduction = a given number of points. (This step is essentially converting the amount into an evidence-based efficacy score.)

**Bioavailability Adjustment:** Modify each component’s score based on the product matrix and form. Apply any reduction factors if the formulation is suboptimal, or full credit if optimal. Sum the adjusted scores for all components; this yields a preliminary subtotal.

**Synergy Bonus:** If the product contains two or more distinct functional ingredients, apply the synergy factor. For example, add a +10% bonus to the subtotal for two different mechanisms, +15% for three, etc. (Exact schema to be determined based on research.) This gives the raw CLCI score for the item.

**Scaling/Normalization:** Optionally, the raw score could be normalized or capped to fit a user-friendly scale (0–100 or similar). For instance, 50 points might correspond to a ~30% LDL reduction (near maximal from foods alone), and scores above this are not practical without medication. The scaling ensures that most real foods fall within a 0–30 range—for example, where <5 is low, 5–15 moderate, and 15+ high capacity. This step also allows for updates over time (if new potent agents are discovered, the scale might be extended).

**Categorization (if needed):** Along with the numeric score, the product could be assigned a category label for simplicity, e.g., low, moderate, or high cholesterol-lowering capacity, based on thresholds (similarly to how the glycemic index is sometimes categorized as low/medium/high GI). For example, >20 points might be “high” (significant cholesterol-lowering effect), 10–20 “moderate”, and <10 “low”.

**Figure 2:**
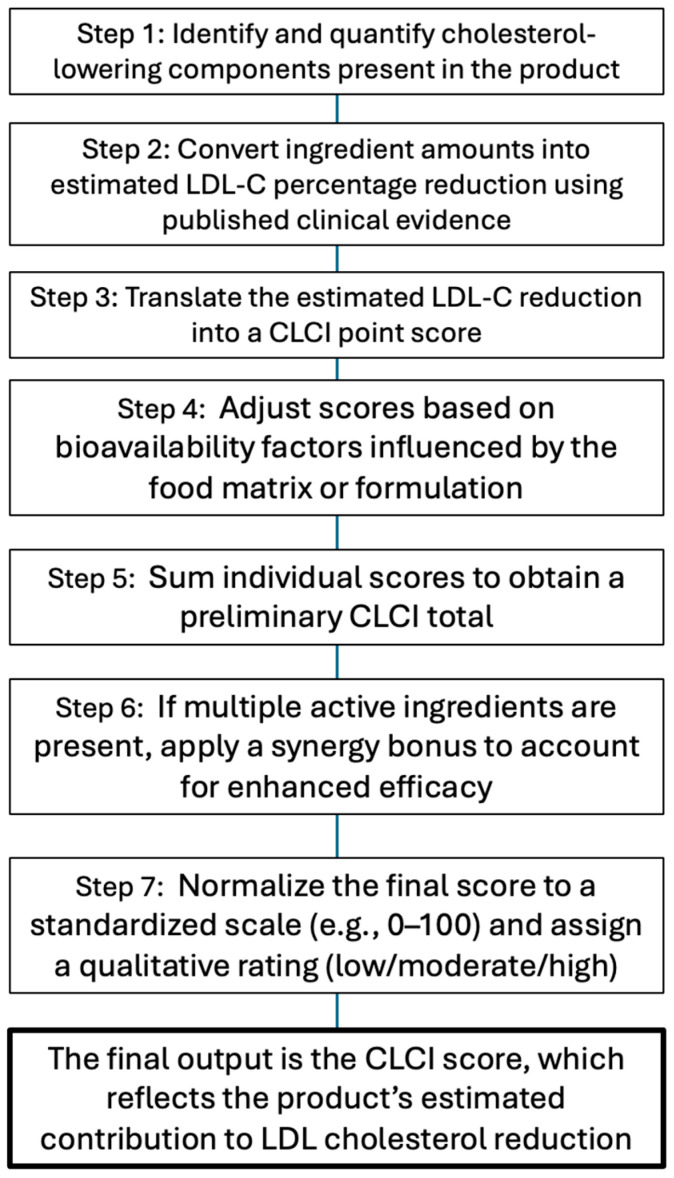
**CLCI calculation flowchart.** Stepwise process for determining the Cholesterol-Lowering Capacity Index (CLCI) of a functional food or supplement. The flowchart outlines the sequential methodology: identifying active components, estimating the LDL-C reduction from clinical data, assigning point scores, applying bioavailability adjustments and synergy bonuses, and standardizing the final score with a qualitative rating.

To demonstrate this, let us consider a hypothetical nutraceutical capsule as an example. Suppose that, per daily dose, it contains 500 mg berberine, 3 mg monacolin K (red yeast rice extract), and 500 mg plant sterols. Based on evidence, we estimate that 500 mg berberine might give a ~10% LDL reduction (half of a full dose ~20%), which may equate to 10 points. Meanwhile, 3 mg monacolin K might give a ~10% reduction (since 5–10 mg yields ~20%, 3 mg is lower), offering another 10 points. Here, 500 mg sterols is a quarter of a full 2 g dose; a 2 g dose gives a ~10% reduction, so 500 mg provides ~2.5%, yielding about 2–3 points. Summing these, the product’s components yield ~22–23 points as a raw score. Now, assume that the capsule form is well absorbed for these ingredients (no major bioavailability penalties). Next, apply the synergy factor: we have three distinct actives (absorption inhibitor, synthesis inhibitor, receptor upregulator), so we can add, for example, a 15% bonus. As 15% of ~23 is ~3.5, the total becomes ~26 points. If our scale indicates that >20 = high capacity, this supplement would be labeled a high-capacity product. We might then normalize this or simply report “CLCI score ≈ 26”. In practice, we might round this and state “CLCI score: 25 (high)”, indicating a strong cholesterol-lowering capacity. Figure 2 illustrates this calculation process.

To illustrate the practical application of the proposed Cholesterol-Lowering Capacity Index (CLCI), Table 2 presents examples of both functional foods and commercially available dietary supplements containing evidence-based cholesterol-lowering ingredients. For each product, the CLCI was calculated using the methodology described earlier, which integrates clinical efficacy data, bioavailability adjustments based on the food matrix or formulation, and synergy factors where multiple mechanisms are involved. These examples demonstrate how the CLCI can differentiate between products with similar ingredients but varying delivery systems, doses, or combinations, offering a standardized framework to evaluate and compare their potential to reduce LDL cholesterol.

### 5.5. Interpreting the CLCI Score

For end-users, the CLCI would be presented in a simple format once calculated. A single number (or a category alongside it) allows quick interpretation. For example, a product might display “Cholesterol-Lowering Index = 15 (Moderate Capacity)”. However, what does a given score mean in practical terms? While the index correlates with the percent LDL reduction, it is not a direct percentage. However, approximate expectations can be communicated. We envisage an interpretation scheme such as the following.

**High CLCI (e.g., ≥20 points):** Indicates a food or supplement that can substantially lower LDL-C. These might be capable of ~15% or greater LDL reductions when used as directed (comparable to strong dietary changes or low-dose drug effects). High-CLCI items will likely contain multiple active ingredients or a very potent single ingredient (like RYR). They could be used as significant adjuncts in cholesterol management, especially for those who cannot take medications.

**Moderate CLCI (e.g., ~10–19 points):** Indicates a moderate cholesterol-lowering effect, perhaps in the order of a 5–15% LDL reduction. Many single-ingredient functional foods fall in this range (e.g., a bowl of oatmeal or a sterol-fortified yogurt might score around 10–15). Incorporating several moderate items in the diet can achieve a meaningful cumulative reduction.

**Low CLCI (e.g., <10 points):** Indicates a small LDL-lowering effect (a few percent). This does not mean that the food is “unhealthy”—it may simply be neutral with respect to cholesterol. For example, an ordinary apple or a probiotic yogurt without added sterols might have a low score. Such foods can still be part of a heart-healthy diet, but they are not major contributors to lowering LDL on their own.

It is important to stress that a low CLCI score does not imply that a food is bad; it may be very healthy in other ways (vitamins, etc.), but it simply does not actively lower cholesterol. The index specifically rates the cholesterol-lowering capacity and not overall nutritional quality. In educational materials, this nuance would be clarified so that consumers or patients understand that they should aim to include enough moderate-/high-CLCI items daily to meet their cholesterol goals, rather than dismissing low-scoring foods entirely.

For practical use, one might set a daily target. For instance, a healthcare provider could advise, “Aim for a total of 20 CLCI points per day through your diet and supplements to achieve a meaningful LDL reduction.” A patient could then mix and match foods, e.g., a sterol-fortified cereal (10 points) + a psyllium fiber drink (5 points) + a serving of almonds (2 points) + a garlic supplement (3 points) = 20 points total for the day. This flexibility empowers individuals to personalize how they reach their “cholesterol-lowering quota” in a way that suits their tastes and lifestyles. It also highlights that relying on one single food might not be enough, echoing the portfolio approach. Conversely, it shows that one high-scoring item can significantly contribute (e.g., a high-CLCI nutraceutical might cover most of the daily target for someone who cannot change their whole diet).

In research, authors of clinical studies could report the CLCI of the diet or intervention tested. For example, if a study uses a combination of sterols and fibers, they might calculate that the regimen had a CLCI of 25, and they observed an X% LDL reduction, facilitating comparisons with another study’s intervention with a similar score. Over time, if the index is validated, one could even predict the expected LDL reduction from the CLCI score with some confidence intervals.

### 5.6. Applications of the CLCI

If established, the Cholesterol-Lowering Capacity Index could have wide-ranging applications.

**Product Labeling and Marketing:** Food manufacturers could include the CLCI on packaging to inform consumers at a glance about heart health benefits. For example, a margarine fortified with plant stanols might display “Cholesterol-Lowering Index: 12 (Moderate)” to signify its benefit. This would empower consumers (especially those monitoring their cholesterol) to compare products quantitatively, as with calories or the glycemic index on some products. It introduces a competitive but health-driven metric, potentially spurring companies to formulate products with higher CLCI scores (i.e., more effective formulations) to gain a market advantage.

**Dietary Planning in Healthcare:** Nutritionists and doctors could use the CLCI to build therapeutic diets. Rather than simply giving general advice (“eat oatmeal, nuts, etc.”), they could aim for a daily or weekly CLCI point total—for instance, “Try to reach at least 15 CLCI points per day in your diet for a mild cholesterol reduction, or ~30 points per day if you need an aggressive reduction.” This transforms abstract advice into a trackable aspect. Patients might even keep a log of their CLCI intake (e.g., “today, I obtained 5 points from breakfast cereal, 8 from a lunch shake, 10 from a dinner spread = 23 points total”). This approach could improve adherence by quantifying progress and giving patients a tangible goal.

**Public Health Guidelines:** Nutritional guidelines might incorporate the concept of the CLCI to enhance current recommendations. For example, rather than simply saying, “eat more foods with soluble fiber and sterols”, guidelines could state, “increase your weekly CLCI points by eating more from the high and moderate groups.” Campaigns could highlight that relying on one “magic” food is not enough—encouraging the public to include a variety (since combining moderate items and obtaining the synergy bonus can lead to a high score, whereas one high item alone might not offset an otherwise unhealthy diet). In essence, it provides a quantitative tool for public education on heart-healthy eating.

**Research and Development:** Scientists developing new nutraceutical extracts or functional foods could use the CLCI framework to estimate the potential impacts of their products and to design studies. If a new ingredient is discovered that lowers LDL by 5%, it could be assigned ~5 points; researchers might then combine it with known ones to create a high-scoring formulation and test it. When publishing, they might report, “this new beverage has a CLCI score of 10 and achieved a 8% LDL-C reduction in our trial”, whereas a competitor product might have five points and a 4% reduction. Over time, as data accumulate, the index itself could be refined (for example, adjusting fiber’s score if new meta-analyses show a different average effect).

In summary, the CLCI aims to be a comprehensive metric that encapsulates a wealth of clinical research into a practical number. It is not meant to replace the nuanced understanding of nutrition science but to complement it by providing a clear, quantitative guide. The development of the CLCI will require a consensus on the scoring algorithm, validation in studies (e.g., do people who consume diets with a higher total CLCI actually see larger LDL drops?), and education to ensure that it is used correctly. If successful, it could become the new means to calculate the anti-cholesterol power of foods, as with the glycemic index for carbohydrates. Embracing combinations of nutraceuticals, accounting for synergy and bioavailability, is likely to be key in the future of nutritional strategies for cholesterol management, and the CLCI is a step toward standardizing this approach.

## 6. Conclusions

Functional foods and nutraceuticals offer a powerful means to lower cholesterol and reduce cardiovascular risks, complementing traditional dietary advice and medications. This review has highlighted how a range of functional ingredients, from plant sterols and soluble fibers to red yeast rice, bergamot, and beyond, can individually improve lipid profiles, and how innovative strategies like ingredient synergies, personalized nutrition, and advanced food technologies are further enhancing their effectiveness. To translate this knowledge into practice, the Cholesterol-Lowering Capacity Index (CLCI) is proposed as a novel, comprehensive metric to evaluate and communicate the LDL-lowering potential of foods and supplements. The CLCI consolidates clinical evidence into a user-friendly score by integrating multiple factors: proven nutraceutical components (and their effective doses), bioavailability in real-world food matrices, and the synergistic boosts from combining ingredients. By doing so, it moves beyond focusing on any single nutrient in isolation and provides a more holistic assessment of a food’s heart health impact.

The CLCI is envisioned as an invaluable tool across various domains. For consumers, it could guide healthier choices and encourage a “portfolio” approach to eating; similarly to the glycemic index supporting blood sugar management, the CLCI would aid in managing cholesterol. For healthcare providers, it offers a concrete way to prescribe diet modifications (e.g., aiming for a target CLCI score) and to articulate the benefits of functional foods in quantitative terms. For the food industry and policymakers, the index could set standards and benchmarks for product development and health claims, ensuring that functional foods deliver meaningful benefits. Importantly, as with any such index, refinement and validation are key next steps. Ongoing research will need to calibrate the scoring system with real-world outcomes and update it as new evidence emerges (for example, if a new meta-analysis finds a certain fiber more effective than expected, its score contribution would be adjusted). In the long term, we encourage studies to explicitly test the CLCI’s predictions—for instance, do people who consistently consume high-CLCI diets experience greater LDL reductions and cardiovascular improvements compared to those with low-CLCI diets?

To facilitate the broader adoption of the CLCI in practice, future efforts should also consider the development of regulatory frameworks or consensus guidelines that support its use. These might include standards for evidence grading, criteria for eligible ingredients and dosages, and alignment with existing structures for health claims in functional foods. Engagement with regulatory agencies, nutrition science organizations, and international standard-setting bodies could be essential to validate and endorse the CLCI as a recognized tool to guide product development, labeling, and dietary recommendations.

The fight against CVD stands to gain from innovative approaches that bridge nutrition science and practical implementation. Functional foods are no longer just curiosities; they are evidence-based options to improve public health. By standardizing the evaluation of these foods through the CLCI and by continuing to design products that are effective, safe, and appealing, we can better integrate nutraceutical strategies into mainstream healthcare. In essence, the CLCI framework encourages combinations of cholesterol-lowering foods, acknowledging that diversity and synergy can yield drug-like benefits, and it provides a common language for stakeholders to discuss and optimize heart-healthy diets. This comprehensive review and the introduction of the CLCI are intended to serve as a reference point and to inspire further developments in cholesterol-lowering functional foods, ultimately contributing to improved cardiovascular outcomes and evidence-informed nutrition policy.

## Figures and Tables

**Table 1:** **Representative studies supporting cholesterol-lowering mechanisms of selected nutraceuticals and dietary strategies.** This table summarizes clinical trials, meta-analyses, and mechanistic studies demonstrating LDL cholesterol reduction through various mechanisms, including the inhibition of intestinal cholesterol absorption, suppression of hepatic synthesis, and replacement of harmful dietary components.

Study (Type and Population)	Key Findings	Ref.
**Inhibition of Cholesterol Absorption**		
Chitosan supplementation—RCT, 90 women with mild hypercholesterolemia (1.2 g/day for 8 weeks) vs. placebo.	Mild LDL-C reduction: Chitosan significantly lowered total cholesterol compared to placebo, with a mild but significant LDL-C decrease (notably in women >60), demonstrating a safe but modest cholesterol-lowering effect.	[11]
Chitosan supplementation—12-week double-blind RCT, 116 obese adults (3.2 g/day) vs. placebo.	LDL-C reduction without absorption marker change: Chitosan treatment led to a significant 5.6% drop in LDL-C vs. placebo (~–8.7 mg/dL, *p* ≈ 0.025). This LDL reduction was not accompanied by expected changes in cholesterol absorption markers, suggesting a modest effect on absorption.	[12]
Sodium alginate (soluble fiber)—Animal study in rats fed high-cholesterol diet.	Increased fecal cholesterol excretion: Soluble alginate formed a gel in the gut, binding dietary cholesterol and bile acids and markedly increasing fecal cholesterol excretion, thereby reducing absorption. Improved glucose tolerance was also observed, highlighting alginate’s potential to combat hypercholesterolemia and diabetes.	[13]
Depolymerized sodium alginate—Controlled trial, 31 healthy women on high-cholesterol diet (4 g/day alginate drink for 3 weeks).	Prevention of dietary cholesterol rise: In the no-alginate group, serum total cholesterol rose from ~178 to 186 mg/dL on a high-cholesterol diet. By contrast, the alginate group’s cholesterol remained stable, preventing the rise in TC seen with high cholesterol intake. The effect was most pronounced in those with higher baseline cholesterol, indicating that alginate inhibited the absorption of dietary cholesterol.	[14]
Green tea catechins (with galloyl groups)—Mechanistic review and animal experiments.	Reduced micellar cholesterol and absorption: Green tea catechins (especially EGCG with a galloyl moiety) were shown to reduce intestinal cholesterol absorption. They decreased cholesterol’s micellar solubility in the gut, leading to increased fecal neutral sterol excretion and lower serum cholesterol levels in animal models. Gallated catechins significantly blocked lymphatic cholesterol uptake in rats, confirming an absorption–inhibition mechanism.	[15]
Green tea catechin (GTC) ingestion—Meta-analysis of 20 RCTs (1415 subjects) with GTC 145–3000 mg/day for 3–24 weeks.	Moderate LDL-C lowering: GTC supplementation produced a significant reduction in LDL-C (~5.3 mg/dL) and total cholesterol (~5.5 mg/dL) compared to controls. There was no significant change in HDL or triglycerides. These results confirm that regular green tea catechin intake yields modest lowering of LDL and total cholesterol.	[16]
Psyllium (soluble fiber) supplementation—Meta-analysis of 28 RCTs (median dose ~10 g/day) in hyperlipidemic adults.	Significant LDL-C reduction: Psyllium fiber reduced LDL-C by ~0.33 mmol/L (~13 mg/dL) versus placebo (*p* < 0.00001). Non-HDL cholesterol was also significantly lowered. This supports psyllium’s FDA-approved role as a cholesterol-lowering adjunct (mechanism: binding bile acids/cholesterol in gut, thereby reducing absorption).	[17]
Oat β-glucan (soluble fiber) intake—Meta-analysis of 28 RCTs (≥3 g/day oat β-glucan).	LDL-C lowering: Adding ≥3 g/day of oat β-glucan led to a mean LDL-C reduction of ~0.25 mmol/L (~9–10 mg/dL) and in total cholesterol of ~0.30 mmol/L (~11–12 mg/dL). HDL and TG were unchanged. Soluble oat fiber increases intestinal viscosity and bile acid excretion, explaining this modest cholesterol reduction.	[18]
Plant sterol/stanol intake—Meta-analysis of 41 RCTs (typically ~2 g/day sterols).	LDL-C lowering: Regular sterol/stanol consumption produced an average LDL-C decrease of ~0.31 mmol/L (~12 mg/dL) vs. placebo (*p* < 0.0001). This ~8–10% LDL reduction (with no effect on HDL) confirms that sterols, by competing with dietary cholesterol for absorption, effectively lower LDL.	[19]
Soy protein + plant sterols—Animal study in hamsters (5-week diet with 0.24% plant sterol esters ± 20% soy protein).	Synergistic cholesterol reduction: Soy protein and sterols each lowered plasma total cholesterol (−9% and −13%, respectively). Combined, they produced a 26% drop in total cholesterol (mainly non-HDL-C)—greater than either alone. The combination markedly increased fecal neutral sterol and bile acid excretion (more than either ingredient alone), indicating a synergistic absorption-blocking effect.	[20]
**Inhibition of Cholesterol Synthesis**		
Omega-3 fatty acids (EPA/DHA)—Dose–response meta-analysis of 90 RCTs (≈72,000 total participants).	Improved plasma lipids (TG ↓, non-HDL-C ↓): Higher omega-3 intake showed a near-linear reduction in triglycerides and non-HDL cholesterol levels. Doses ≥2–3 g/day were especially effective in hyperlipidemic individuals. (LDL effects were dose-dependent; overall non-HDL improvement suggests net benefit.) Omega-3s lower VLDL production and enhance clearance, aligning with the inhibition of hepatic lipid synthesis/secretion.	[21]
**Replacement of Harmful Nutrients/Dietary Patterns**		
Polyunsaturated vs. saturated fat—Mechanistic insights from metabolic studies.	PUFAs yield less LDL: Replacing saturated fats with polyunsaturated fatty acids causes the liver to burn PUFAs as energy (ketone production) instead of producing VLDL. Consequently, fewer VLDL remnants are left to form LDL. This explains why diets high in PUFAs (e.g. plant oils, nuts, fatty fish) consistently lower serum LDL-C compared to SFA-rich diets.	[22]
**Replacement of Nutrients/Diet Patterns**		
Replacing SFAs with PUFAs in diet—Randomized crossover trial, 17 healthy adults (3-day diets of high SFA vs. high PUFA, with washout).	Rapid cholesterol improvement: Switching from a butter-rich diet to a high-PUFA diet (using plant oils) for just 3 days lowered total cholesterol by ~8% (*p* = 0.002). The PUFA diet also increased gut Lachnospiraceae and other beneficial microbes, which correlated with the cholesterol drop. This suggests that improving fat quality (replacing animal/saturated fat with plant PUFAs) quickly reduces cholesterol levels, potentially via gut microbiome interactions.	[23]
Soy protein vs. animal protein—Meta-analysis of 46 trials (median 25 g/day soy protein for 6 weeks).	Small but significant LDL-C reduction: Across studies, replacing some animal protein with soy protein led to a ~3–4% decrease in LDL-C (~5 mg/dL) and a ~6 mg/dL drop in total cholesterol. While modest, this supports recommendations to increase plant proteins (like soy) for better cholesterol profiles. Soy’s benefit is partly due to displacing saturated fat from animal foods and inherent compounds (isoflavones, fibers), promoting cholesterol clearance.	[24]
Traditional Japanese vs. Western diet—Cross-sectional analysis of Japanese adults (National Health and Nutrition Survey).	Diet pattern influences cholesterol: A “Westernized” dietary pattern (higher in meats and fats) was associated with higher total and LDL cholesterol levels in both men and women. In contrast, those with higher adherence to a traditional Japanese diet (fish, soy, vegetables, etc.) tended to have lower serum cholesterol. These data illustrate that replacing Western diet elements with more traditional/plant-based foods correlates with better lipid profiles.	[25]

**Table 2:** **Functional foods and supplements with cholesterol-lowering nutraceuticals (and CLCI scoring).** Each example includes its active ingredients, mechanism of action, clinical LDL-C reduction, bioavailability or formulation notes, presence of synergistic components, and the calculated Cholesterol-Lowering Capacity Index (CLCI) with a rating, as proposed in the manuscript.

Product (Brand)	Active Ingredient(s) (per Serving)	Mechanism(s) of Action	LDL-C Reduction (%)	Bioavailability/Formulation	CLCI Score (Base + Adj + Synergy)	CLCI Rating
Plant sterol-fortified spread (Benecol®)	Plant stanol esters (~2 g stanols/day via spread)	Inhibits cholesterol absorption in gut (stanols compete with dietary cholesterol in micelles)	~7–10% LDL-C reduction with ~2 g/day plant stanols	Fat-based spread matrix aids sterol solubility and micelle incorporation, delivering full efficacy (vs. low-fat vehicles)	≈10 points (base ~10 for 2 g; optimal fat matrix = no bioavailability penalty; no synergy)	Moderate
Plant sterol-fortified yogurt drink (Danacol®)	Plant sterol esters (~1.6–2 g sterols per yogurt drink)	Inhibits intestinal cholesterol absorption (sterols displace cholesterol in micelles)	~7–10% LDL-C reduction (daily sterol drink regimen)	Low-fat dairy matrix can slightly reduce sterol efficacy; often uses microencapsulated sterols for better dispersion in yogurt/juice	≈9 points (base ~10; ~80–90% effective in low-fat matrix → slight score reduction; no synergy)	Low (borderline moderate)
Oatmeal (Quaker® Oats)	Oat β-glucan soluble fiber (~3 g β-glucan per bowl of oatmeal)	Binds bile acids in gut with viscous gel, reducing cholesterol reabsorption and increasing excretion	~5–7% LDL-C reduction with ~3 g/day oat β-glucan	Must be hydrated/cooked to form viscous gel; high-molecular-weight β-glucan yields greater effect	≈5 points (base ~5 for 3 g; no bioavailability penalty if prepared properly; no synergy)	Low
Psyllium husk supplement (Metamucil®)	Psyllium soluble fiber (~10 g/day from fiber supplement)	Forms gel that sequesters bile acids, inhibiting cholesterol absorption in the intestine	~5–10% LDL-C reduction at ~10 g/day psyllium	Requires adequate water intake for gel formation; taken with meals for best effect (viscosity critical to binding bile)	≈5 points (base ~5 for 10 g; full efficacy if properly hydrated; no synergy)	Low
Soy protein food (e.g., soy milk/tofu)	Soy protein (~25 g, from ~2–3 servings of soy food)	Replaces high-saturated-fat animal protein (lowering hepatic cholesterol synthesis); soy peptides/isoflavones directly improve LDL clearance in liver	~3–7% LDL-C reduction with ~25 g/day soy protein	Requires daily intake of ~25 g; typically consumed via soy foods or protein shakes as part of diet (no special formulation needed for activity)	≈4 points (base ~3–5 for 25 g; no bioavailability issues for protein; no synergy)	Low
Red yeast rice extract (RYR supplement)	Monacolin K (~5–10 mg/day from red yeast rice)	Inhibits HMG-CoA reductase (statin-like blockade of cholesterol synthesis in liver)	~15–25% LDL-C reduction at 5–10 mg/day monacolin K	Oral supplement provides active lovastatin analog; ensure standardization (potency can vary); avoid high heat (cooking degrades monacolin)	≈18 points (base ~15–20 for effective dose; no absorption penalty when taken with food; no synergy)	High
Berberine capsule (nutraceutical)	Berberine HCL (~1000 mg/day, typically 2 × 500 mg capsules)	Upregulates hepatic LDL receptors (enhances LDL clearance); also modestly inhibits cholesterol synthesis	~10–15% LDL-C reduction at ~1 g/day dose (meta-analyses)	Moderate oral bioavailability; usually given in divided doses (e.g., 500 mg twice daily) to maintain levels; new formulations (e.g., lipid carriers) may improve absorption	≈10 points (base ~10 for 1 g; standard capsule form = no significant bioavailability adjustment; no synergy)	Moderate
Bergamot citrus extract (Citrus bergamia supplement)	Bergamot polyphenol extract (~500 mg/day, high-flavonoid citrus extract)	Multiple actions: inhibits HMG-CoA reductase (reduces cholesterol synthesis) and ACAT; upregulates LDL receptors (increasing LDL uptake); possibly reduces cholesterol absorption	~8–15% LDL-C reduction observed at 500–1500 mg/day doses	Taken as standardized capsule; polyphenol absorption is moderate—best if formulation enhances bioavailability (e.g., phytosome); must protect extract from high heat/light to preserve active flavonoids	≈10 points (base ~10 for standard dose; assume no formulation penalty with optimized extract; no synergy)	Moderate
Garlic extract (Kyolic® aged garlic)	Aged garlic extract (~1000 mg/day, supplying stable organosulfur compounds)	Mild inhibition of hepatic cholesterol synthesis (garlic’s allicin and metabolites downregulate HMG-CoA reductase activity)	~5% LDL-C reduction on average (up to ~10% in some studies with high-dose garlic)	Active allicin is unstable; aging process yields S-allyl-cysteine (more stable and bioavailable); daily supplementation needed for sustained effect	≈4 points (base ~3–5 for ~1 g; no significant bioavailability issues with aged extract; no synergy)	Low
Armolipid Plus® (multicomponent supplement)	Red yeast rice (monacolin K 3 mg); berberine 500 mg; policosanol 10 mg; folic acid 0.2 mg; astaxanthin 0.5 mg; coenzyme Q10 2 mg	Combination of multiple mechanisms: monacolin K + policosanol inhibit cholesterol synthesis; berberine upregulates LDL clearance; astaxanthin provides antioxidant support; folic acid reduces homocysteine (cardio-protective)	~20% LDL-C reduction observed (multi-ingredient synergy comparable to low-dose statin effect)	Oral tablet formulation; contains fat-soluble components (policosanol, astaxanthin, CoQ10)—advised to take with food for optimal absorption; no major bioavailability issues reported for the combination	≈26 points (base ~22 combined from ingredients; +15% synergy bonus for 3 distinct mechanisms → ~26 total)	High
